# Efficacy of surgical repair for the functional restoration of injured facial nerve

**DOI:** 10.1186/s12893-021-01049-x

**Published:** 2021-01-08

**Authors:** Li Li, Zhaomin Fan, Haibo Wang, Yuechen Han

**Affiliations:** Department of Otology Surgery, Shandong Provincial ENT Hospital, Cheeloo College of Medicine, Shandong University, No. 4 Duanxing West Road, Huaiyin District, Jinan, 250021 People’s Republic of China

**Keywords:** Facial nerve injury, Surgical repair, Electromyogram

## Abstract

**Background:**

Early surgical repair to restore nerve integrity has become the most commonly practiced method for managing facial nerve injury. However, the evidence for the efficacy of surgical repair for restoring the function of facial nerves remains deficient. This study evaluated the outcomes of surgical repair for facial nerve lesions.

**Methods:**

This retrospective observational study recruited 28 patients with the diagnosis of facial nerve injury who consecutively underwent surgical repairs from September 2012 to May 2019. All related clinical data were retrospectively analyzed according to age, sex, location of the facial nerve lesion, size of the facial nerve defect, method of repair, facial electromyogram, and blink reflex. Facial function was then stratified with the House-Brackmann grading system pre-operation and 3, 9, 15, and 21 months after surgical repair.

**Results:**

The 28 patients enrolled in this study included 17 male and 11 female patients with an average age of 34.3 ± 17.4 years. Three methods were applied for the repair of an injured facial nerve, including great auricular nerve transplantation in 15 patients, sural nerve grafting in 7 patients, and hypoglossal to facial nerve anastomosis in 6 patients. Facial nerve function was significantly improved at 21 months after surgery compared with pre-operative function (*P* = 0.008). Following surgical repair, a correlation was found between the amplitude of motor unit potential (MUP) and facial nerve function (r = -6.078, *P* = 0.02). Moreover, the extent of functional restoration of the facial nerve at 21 months after surgery depended on the location of the facial nerve lesion; lesions at either the horizontal or vertical segment showed significant improvement(*P* = 0.008 and 0.005), while no functional restoration was found for lesions at the labyrinthine segment (*P* = 0.26).

**Conclusions:**

For surgical repair of facial nerve lesions, the sural nerve, great auricular nerve, and hypoglossal-facial nerve can be grafted effectively to store the function of a facial nerve, and MUP may provide an effective indicator for monitoring the recovery of the injured nerve.

## Background

Facial nerve injury is a common clinical entity, occurring as a result of mechanical, chemical, or ischemic damage caused either by trauma, tumor, or iatrogenic injury. In as many as 60%–75% of patients with facial palsy, the cause is idiopathic paralysis or Bell’s palsy [1–4]. Other common etiologies are various traumas with temporal bone fractures, cholesteatoma of the middle ear, and schwannomas of the facial or the vestibular nerve. Sunderland classified significant facial nerve injury according to five degrees [5]. First-degree injury refers to an undisrupted nerve with neurapraxia produced by increased intraneural pressure from external compression; second-degree injury involves Wallerian degeneration of the nerve axons but leaves the surrounding membranes intact; and the third- through fifth-degrees of injury reflect partial or complete transection of the nerve with the loss of endoneurial, perineurial, and epineurial tubes, respectively.

The management of the injured facial nerve depends upon the etiology of the injury. In general, the primary paradigm for managing damaged facial nerve has been early microsurgical repair and restoration of nerve continuity [6, 7]. When a facial nerve is transected, direct cooptation is the best choice. However, if the primary repair is not feasible, a nerve autograft to bridge the transected nerve should be utilized as long as the motor end plates are still intact. Moreover, the indication for surgery also depends on the severity of the nerve lesion. Non-degenerative neuropraxia from blunt trauma will not need surgical reconstruction, whereas disruption leading to degenerative neurotmesis likely does require surgical treatment. Finally, any tumor in the course of the facial nerve from the brainstem to the periphery can cause facial palsy, or surgical treatment of the tumor might be the cause of facial palsy. In such circumstances, typically surgery of the primary disease is combined with surgical reconstruction of the facial nerve.

To date, numerous publications in the literature have focused on the procedural details of the most commonly applied operations, including direct repair, cable nerve grafting, and nerve crossover techniques for facial nerve repair and restoration. After the repairs and reconstruction, a House-Brackmann grade III is the best possible result with persistent residual weakness and synkinesis on the repaired and reconstructed facial nerve attributed to factors such as older age at the time of repair, long grafts, and extended delay between time of injury and repair, which all reduce the functional outcome of the repair and reconstruction. Here we summarize our experiences in 28 patients who underwent facial nerve repair and construction and further investigate the efficacy of our surgical repair approach for restoring the function of a transected facial nerve.

## Methods

The hospital ethical committee approved this observational study and waived the requirement for informed consent (Approval number: XYK20191201). 28 consecutive patients were enrolled in this study from September 2012 to March 2019 at our hospital. All clinical data were collected from the medical records of the 28 patients, including the location of the facial nerve lesion, the defect of the facial nerve, the duration of facial paralysis before surgical intervention, the pathology of the lesion, the method of nerve repair, and the facial electromyogram.

Facial nerve function was evaluated via the House–Brackmann grading system pre-operation and 3, 9, 15, and 21 months following surgery. The patients were routinely followed up every 3 months after nerve repair until no further improvement of facial function was observed. Follow-up at 21 months after the operation was set as the final follow-up for the assessment of facial nerve function.

### Surgical technique

For facial nerve that was completely transected, the proximal and distal portions of the injured nerve must be first identified for bettering reconstruction surgery. Facial nerve repair with cable grafting of the sural nerve or great auricular nerve, or a hypoglossal-facial nerve transfer was performed immediately after characterizing the lesion on the facial nerve. The greater auricular nerve was utilized to repair cases in which the defect of the facial nerve was less than** 7** cm in length, while the sural nerve was usually employed to bridge a gap of longer length. When the proximal stump of the nerve at the brainstem cannot be identified, the motor nerve, such as hypoglossal to facial anastomosis, might be an optional procedure to restore continuity and function of facial nerve [8, 9].

### Functional assessment

The House–Brackmann grading system was applied to assess the function of the repaired facial nerve according to six grades (grade I = normal function to grade VI = complete paralysis plus gross asymmetry at rest) [10]. A standardized clinical examination included analysis of voluntary movements including frowning, eye closure, nose wrinkling, smile, open mouth, teeth showing, and lip pursing with electromyographic (EMG) evaluation. EMG was recorded on four muscles including the frontal muscle, orbicularis oculi muscle, zygomatic muscle, and orbicularis oris muscle. The post-operative activity of facial muscle was studied by still photography of the patients (at rest, smiling, eye closure, eyebrow-raising, and frowning).

### Statistical analysis

Statistical analyses of the data were performed using SPSS 19.0 software. The amplitude and duration of the motor unit potential (MUP) were analyzed with the repeated analysis of variance (ANOVA). To compare the facial function pre-operation and at 21 months post-operation, changes in the House–Brackmann grading scores were tested with the paired t-test. The Spearman correlation analysis was performed to examine the correlation between House–Brackmann grade pre-operation and at 21 months after operation as well as the correlation between the duration of facial nerve paralysis and House–Brackmann grade pre-operation. The changes in facial nerve function before and after operation according to the House-Brackmann grading system for facial nerve lesions at different locations were compared with grouped t-test. The significance level was set at *P* < 0.05. Five patients with House-Brackmann grade VI were processed as missing data due to failing to receive a read out from the EMG machine.

## Results

The etiologies of facial nerve lesions in the patients who underwent surgical repair of facial nerves included facial nerve neurilemmoma (16 cases), facial nerve hemangioma (2 cases), facial neurofibroma (1 case), glomus jugulare tumor (1 case), mastoid hamartoma (1 case), facial nerve burn-injured (1 case), facial nerve mechanical injury (1 case), iatrogenic facial nerve injury (1 case), and petrous apex cholesteatoma (4 cases) (Table 1). The surgical approaches applied for the patients who suffered from facial nerve resection included great auricular nerve transplantation in 15 cases, sural nerve graft in 7 cases, and hypoglossal to facial nerve anastomosis in 6 cases (Table 1). All the repairs were performed with atraumatic handling of tissues, exact end-to-end anastomosis, and tension-free closure. The great auricular and sural nerve graft was reversed, and the distal end was coapted to the donor facial nerve branch. All of the patients with hypoglossal-facial transfers underwent classic end-to-end hypoglossal-facial anastomosis [11].

**Table 1:** Demography of time of facial paralysis, etiology, facial nerve function, surgical methods, and synkinesis

No.	Time of facial paralysis preoperation (month)	Etiology of facial nerve lesions	Facial nerve function (House–Brackmann Grading)		Surgical methods	Location of lesion	Synkinesis (21 months postoperation)
			Preoperation	21 months postoperation			
1	30	Facial nerve neurilemmoma	IV	IV	Great auricular nerve transplantation	Vertical segment	Yes
2	7	Facial nerve hemangioma	III	III	Hypoglossal to facial nerve anastomosis	Labyrinthine segment + Geniculate Ganglion	Yes
3	14	Facial neurofibroma	IV	III	Great auricular nerve transplantation	Vertical segment	Yes
4	0	Glomus jugulare tumors	I	III	Sural nerve graft	Horizontal segment + vertical segment	Yes
5	28	Mastoid hamartoma	V	IV	Great auricular nerve transplantation	Horizontal segment + vertical segment	Yes
6	9	Facial nerve neurilemmoma	II	III	Great auricular nerve transplantation	Vertical segment	Yes
7	4	Facial nerve mechanical injury	VI	III	Great auricular nerve transplantation	Horizontal segment	Yes
8	14	Facial nerve neurilemmoma	IV	III	Sural nerve graft	Horizontal segment + vertical segment	Yes
9	0	Facial nerve neurilemmoma	I	III	Great auricular nerve transplantation	Pyramidal segment	Yes
10	11	Facial nerve neurilemmoma	V	III	Great auricular nerve transplantation	Horizontal segment + vertical segment	Yes
11	19	Facial nerve neurilemmoma	IV	IV	Sural nerve graft	Horizontal segment + vertical segment	Yes
12	6	Facial nerve burn-injured	VI	III	Great auricular nerve transplantation	Horizontal segment + vertical segment	Yes
13	11	Facial nerve hemangioma	IV	III	Great auricular nerve transplantation	Horizontal segment	Yes
14	15	Facial nerve neurilemmoma	V	III	Great auricular nerve transplantation	Vertical segment	Yes
15	7	Iatrogenic facial nerve injured	V	III	Great auricular nerve transplantation	Pyramidal segment	Yes
16	17	Facial nerve neurilemmoma	V	IV	Hypoglossal to facial nerve anastomosis	Labyrinthine segment + horizontal segment	Yes
17	22	Facial nerve neurilemmoma	III	III	Great auricular nerve transplantation	Vertical segment	Yes
18	28	Facial nerve neurilemmoma	V	IV	Sural nerve graft	Horizontal segment + vertical segment	Yes
19	29	Facial nerve neurilemmoma	V	IV	Great auricular nerve transplantation	Vertical segment	Yes
20	39	Facial nerve neurilemmoma	IV	IV	Great auricular nerve transplantation	Horizontal segment + vertical segment	Yes
21	44	Facial nerve neurilemmoma	V	V	Sural nerve graft	Horizontal segment + vertical segment	Yes
22	24	Facial nerve neurilemmoma	V	IV	Sural nerve graft	Horizontal segment + vertical segment	Yes
23	12	Facial nerve neurilemmoma	V	III	Great auricular nerve transplantation	Vertical segment	Yes
24	30	Petrous apex cholesteatoma	V	IV	Hypoglossal to facial nerve anastomosis	Labyrinthine segment + horizontal segment	Yes
25	31	Facial nerve neurilemmoma	VI	V	Hypoglossal to facial nerve anastomosis	Labyrinthine segment + horizontal segment	Yes
26	29	Petrous apex cholesteatoma	VI	IV	Sural nerve graft	Horizontal segment + vertical segment	Yes
27	70	Petrous apex cholesteatoma	VI	VI	Hypoglossal to facial nerve anastomosis	Labyrinthine segment + horizontal segment	Yes
28	122	Petrous apex cholesteatoma	VI	VI	Hypoglossal to facial nerve anastomosis	Labyrinthine segment + horizontal segment + vertical segment	Yes

Table 2 presents the results of the functional evaluation on the damaged facial nerve according to the House-Brackmann grading system. Among our 28 patients, 25 underwent the repair and reconstruction of a damaged facial nerve with a House-Brackmann grade of III or above. The remaining 3 patients had preoperative House-Brackmann scores of I or II and they underwent resection of tumors encasing the facial nerve (one glomus jugulare and two facial neurilemmomas). Because the paralysis was caused intraoperatively, these patients did not have preoperative House-Brackmass scores reflective of their pathologies.

**Table 2:** Facial nerve function according to House–Brackmanngrading system

Facial nerve function (House–Brackmann grade)						
	I	II	III	IV	V	VI
Pre-operation	2	1	2	6	11	6
3 months post-operation			1	7	13	7
9 months post-operation			8	11	5	4
15 months post-operation			10	11	4	3
21 months post-operation			14	10	2	2

Facial EMG indicated that the duration of motor unit potential (MUP) was longer at the side with a facial nerve lesion than at the side without in 23 patients, and the amplitude of MUP was lower on the affected sidein 23 cases (Table 3). Additionally, the MUP could not be recorded in 5 cases. The latent periods of the R1 and R2 wave of the affected side prolonged in 11 cases in the Blink Reflex test, whereas these waves had disappeared in 17 cases. The interval between onset of the palsy and facial nerve reconstruction surgery ranged from 0 to 122 months.

**Table 3:** Changes in the amplitude and duration of MUP. Data are presented as mean (SD).*P < 0.05 compared with pre-operation

	Amplitude, mv	*P*	Duration, ms	*P*
Pre-operation	0.324 (0.159)		7.61 (2.43)	
3 months post-operation	0.581 (0.202)	0.321	7.64 (2.52)	0.397
9 months post-operation	0.981 (0.362)	0.012*	8.13 (3.02)	0.422
15 months post-operation	1.172 (0.355)	0.003*	8.58 (2.47)	0.004*
21 months post-operation	1.204 (0.41)	0.001*	6.87 (2.01)	0.021*

**Fig. 1:**
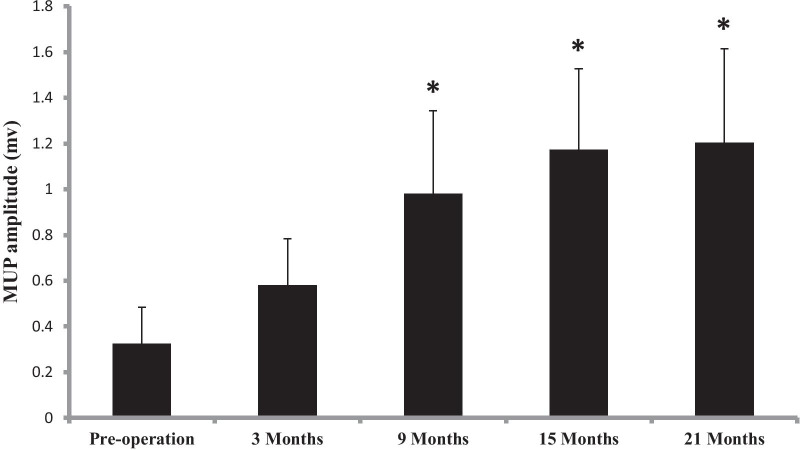
Postoperative changes in the amplitude of the MUP.*P < 0.05 compared with pre-operation

**Fig. 2:**
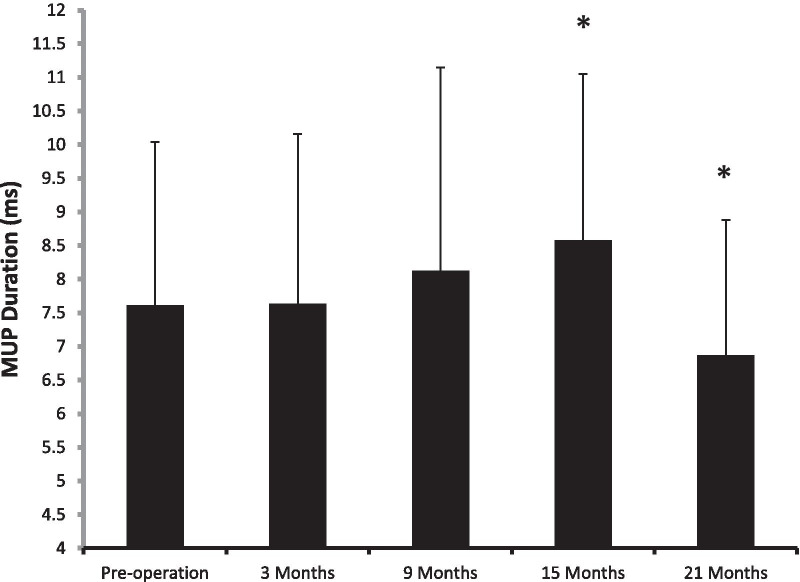
Postoperative changes in the duration of the MUP. *P < 0.05 compared with pre-operation

The first facial movements were observed clinically after 4.76 ± 1.85 months, and the maximal movement was seen after 18.13 ± 5.30 months. On electromyography, the first regeneration potentials were seen after 4.06 ± 1.72 months, and the first MUPs were recorded three months after the repair operations. The postoperative MUP amplitudes of the facial muscles increased significantly compared with the preoperative values and gradually reached peak values 9–21 months after the operation (*P* = 0.012, 0.003, and 0.001;Table 3 and Fig. 1). The MUP duration continuously increased from 3–15 months post-operation but decreased at 21 months after the surgeries. The post-operative MUP duration at 15 months was significantly longer than that pre-operation (*P* = 0.004), while that at 21 months post-operation showed a statistical decrease in comparison with that before the surgeries (*P* = 0.021; Table 3 and Fig. 2).

**Fig. 3:**
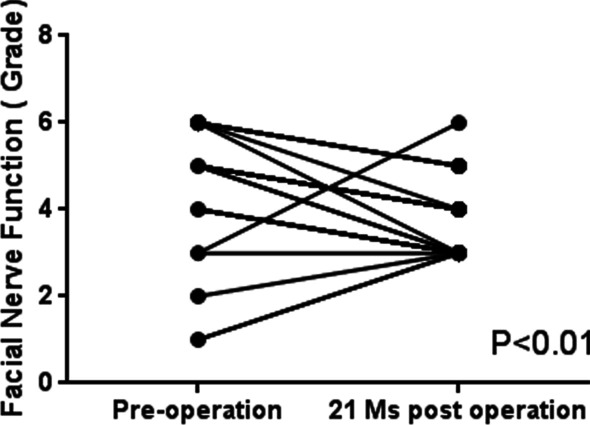
Changes of facial nerve function between pre-operation and 21 months post-operation

At 21 months post-operation, the latent periods of the R1 and R2 waves on the injured side were found to be prolonged in 26 cases, whereas the waves were totally disappeared in 2 patients. The time periods for showing the maximal recovery of the facial muscle movement are as followed:15 months after the operation for 20 patients and 21 months post-operation for 8 patients. At 21 months post-surgery, the function of the repaired facial nerve possessed House-Brackmann grade III in 14 patients, grade IV in 10 patients, grade V in 2 patients, and grade VI in 2 patients (Table 2). Moreover, the facial nerve function according to the House-Brackmann grading system was significantly improved at the 21 months post-operation compared with that pre-operation (*P* = 0.008; Fig. 3).

**Fig. 4:**
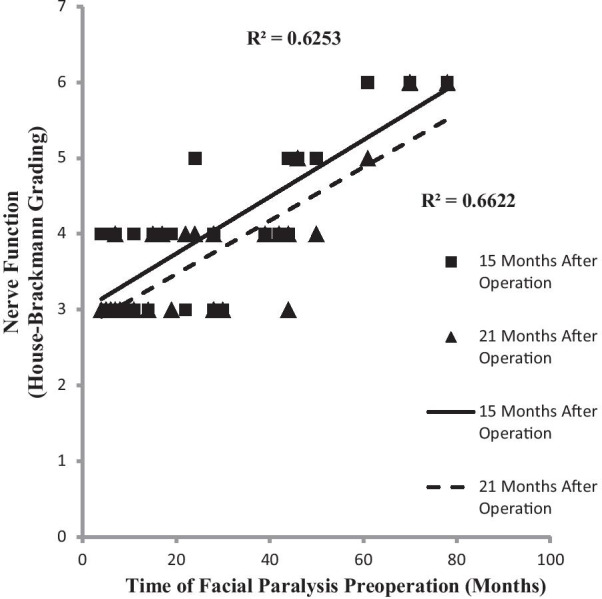
Correlation between the pre-operative duration of facial paralysis and nerve function at 15 and 21 months post-operation (both *P* < 0.05)

**Fig. 5:**
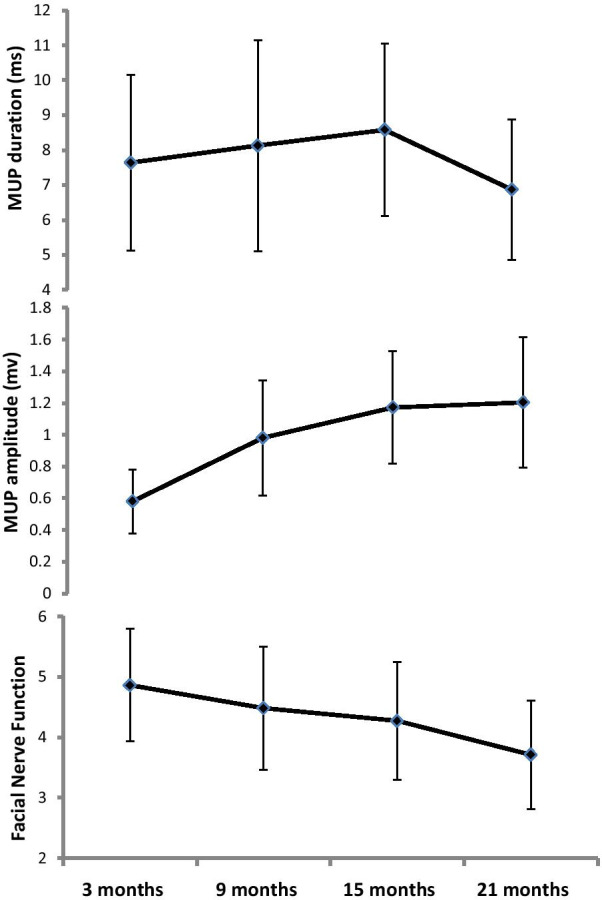
Correlations between the MUP amplitude and facial nerve function (*P* < 0.05)

A correlation between pre-operative and post-operative facial nerve function could not be established in this study (*P* > 0.05); however, a correlation was observed between the length of time from injury to repair and function recovery at 15 months(r = 0.6253, *P* < 0.01) and 21 months after operation (r = 0.6622, *P* < 0.01; Fig. 4). The longer the pre-operative period of the facial paralysis persisted, the worse the functional recovery of the repaired facial nerve was at 15 and 21 months post-operation. Additionally, there was no correlation between the amplitude of MUP at pre-operation and the functional recovery of facial nerve at 15 (*P* = 0.27) and 21 months after operation (*P* = 0.19). However, the correlation between the post-operative MUP amplitude and the functional recovery of facial nerve was found (r = -6.078, *P* = 0.02). Specifically, the MUP amplitude gradually increased with time at later follow-ups, and the function of the facial nerve improved following the same pattern (Fig. 5). This study did not show the correlation between the duration of post-operative MUP and the improvement of facial nerve function (*P* = 0.33); instead, when the duration of MUP transitioned from high to low at 21 months post-operation (compared with the duration pre-operation, *P* = 0.029), facial nerve function showed obvious improvement (Fig. 5).

**Fig. 6:**
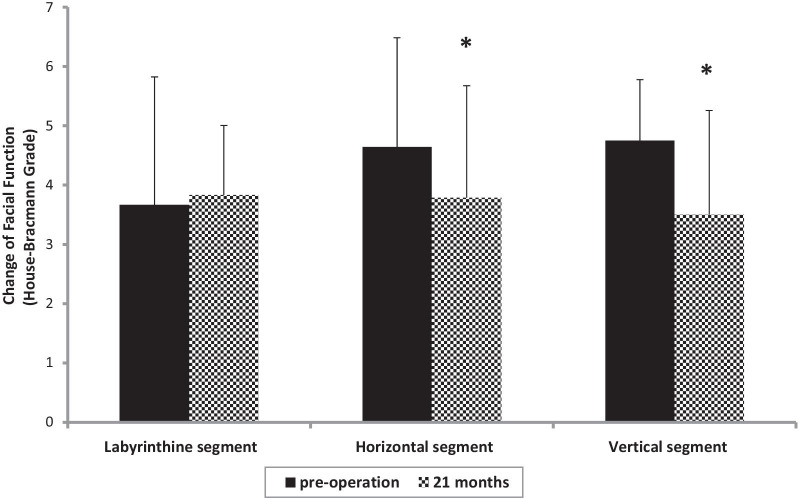
Changes in facial nerve function for lesions at different locations from pre-operation to 21 months after facial repair surgery (*P < 0.05)

Based on the starting location, the facial nerve lesions were classified into three categories: labyrinthine segment, horizontal segment, and vertical segment. According to the House-Brackmann grading, the recovery of facial nerve function at 21 months post-operation was different depending on the location of the facial nerve lesion. Facial nerves with lesions at either horizontal or vertical segment showed significant improvement in postoperative functional recovery vs. pre-operative function(*P* = 0.008, and 0.005). However, no change in facial nerve function was observed for lesions located at the labyrinthine segment (*P* = 0.26; Fig. 6).

## Discussion

Facial nerve injury often occurs in cases where the facial nerve is firmly attached to the lesion and the nerve either hampers surgical access or acts as an impediment to the procedure in otologic surgery. After the facial nerve lesion has been resected, inevitably, the facial nerve is interrupted or partially damaged. However, the best approach to repair the damaged nerve and restore its function still remains uncertain, especially when the gap between the two ends of the transected nerve is greater than 5 mm, which had been evidenced to lead to an impossibility in spontaneous regeneration of the axon [12], and also made the direct neurorhaphy unrealistic [13, 14].

Previous studies showed that the autologous graft might be a good method for peripheral nerve reconstruction [14–16]. However, the efficacy of this grafting for restoring facial nerve function remained undefined. Some studies reported that the sensory nerves, such as the sural nerve or great auricular nerve, could be harvested for autografting [16, 17]. Clinically, facial muscle atrophy could partly be avoided by the hypoglossal-facial nerve bridge [18]. In present study, we found that the hypoglossal nerve bridge did improve patients’ House–Brackmann grade from VI to III [19], suggesting the advantage of this bridging technique.

Facial nerve cable grafting was performed in present case series with the sural nerve, great auricular nerve, or hypoglossal-facial nerve transfer, in order to treat 28 facial nerve injuries of different House-Brackmann grades. Repair was performed at all anastomoses by use of atraumatic handling of tissues, exact end-to-end anastomosis, and tension-free closure. The great auricular and sural nerve grafts were all reversed so that the distal end of the graft is attached to the proximal end of the donor nerve. More importantly, this study demonstrated that the post-operative facial nerve function gradually improved, reaching peak functional restoration at 21 months post-operation. Therefore, cable grafting with the sural nerve or great auricular nerve and hypoglossal-facial nerve transfer may be effective options for surgical repair of facial nerve damage.

Moreover, following the repair surgery, MUPs re-appeared before the first clinical facial movements in our patients, which is consistent with the finding of Flasar et al. [20]. The MUP amplitude increased gradually along with the improvement of facial nerve function. Additionally, the MUP duration showed a crescendo–decrescendo pattern with a turning point at 21 months post-operation. The MUP duration reflects the consistency of the electric activity of nerve fibers [21], and an increased MUP duration reflects less consistent electrical activity [22, 23].After the repair surgeries, the spreading velocity of the electric signal in facial nerve fibers differed due to different degree of the nerve damage. The gradual rise and then gradual fall of the MUP duration indicated that the function of the damaged nerve fiber recovered gradually, and the decrease in duration means electric broadcasting of the repaired nerve was obviously increased. Taken together, our study suggested the MUP could be counted as an effective indicator for monitoring the functional recovery of the repaired facial nerve.

Using MUP for monitoring the functional recovery of damaged nerve was rare in previous literature. An earlier study measured MUP through recording the changes in EMG over time [24] to evaluate facial nerve function. The results of this study showed that electromyographic activity occurred before the facial movement, which was consistent with our findings in our study. Additionally, we observed persistent improvement of facial nerve function through 21 months post-operation. Therefore, we recommend that MUP of facial nerve function should be continuously monitored for more than 21 months post-operation.

In the present study, the location of the nerve injury was shown to be a critical factor that significantly impacted the outcome of the surgical repair. Facial nerves with an injury close to the proximal end of the nerve had worse functional recovery after the repair surgery, whereas nerves with the injury close to the distal end had better functional recovery. Additionally, we observed relatively poor results for lesions in the labyrinthine segment, which echoes the previous findings on the surgical repair of other peripheral nerve injuries [10, 25]. One possible explanation is that the component of nerve differs between the proximal and distal segments. The facial nerve possesses sensory and motor nerve fibers divided from its horizontal and vertical segments, but the labyrinthine segment lacks branches, which means more mixed nerve fiber crossing over with each other in the proximal segment than in the distal endings of the facial nerve fiber, and hypothetically, the crossover growth could be a barrier to the functional restoration of the repaired facial nerve.

Anatomically, the labyrinthine segment was thinner than the horizontal and vertical segments of facial nerves, making the greater auricular nerve and sural nerve transplants difficult to implant in some cases. Thus, hypoglossal–facial anastomosis can be fashioned when the proximal facial nerve has been resected, only if the distal nerve and facial musculature are viable. In our study, all hypoglossal-facial anastomosis patients possessed a labyrinthine segment lesion. Greater auricular nerve and sural nerve grafts were also performed in some cases of labyrinthine segment lesion. The results of our study showed that recovery of the labyrinthine segment was worse than that of horizontal segment and vertical segment, suggesting the site of the lesion along the facial nerve should contribute to the choice of surgical method and influence the clinical outcome of the repair. The reason may be related to the location of the injury and surgical method.

The present study further established the correlation between the duration of facial paralysis after injury and the grading of nerve function at 15 and 21 months after operation. With a shorter facial paralysis time, better recovery of facial nerve function was observed. Thus, early surgical repair was associated with better prognosis [26, 27]. A previous study observed that patients who underwent acute hypoglossal-facial anastomotic repair (0–14 days from injury) were more likely to achieve nerve function with House-Brackmann grade ≤ 3 compared to those had delayed repair (average time from injury to reanimation more than 6 months) [28, 29].

Additionally, some clinical evidence showed that some extent of functional recovery could be achieved with facial nerve repair even after a more than 2-year delay [14, 27]. Our study also found that even the patients with long-standing facial nerve injuries might still benefit from the repair surgery. One of the reasons is that nerve repair can effectively prevent muscle atrophy [30], even if the facial nerve function does not recover well after facial nerve transplantation or hypoglossal to facial nerve bridging.

## Conclusions

Restoration of facial nerve function after injury has been a long-standing challenge. This study demonstrated that surgical repair with either great auricular nerve transplantation, or sural nerve graft, or hypoglossal to facial nerve anastomosis was effective for repairing the damaged facial nerve and restoring its function. Furthermore, facial nerve function test demonstrated significant improvement at 21 months post-operation compared with that pre-operation. Finally, the amplitude and duration of the MUP were shown to be effective indicators for monitoring the effect of facial nerve reconstruction in the patients with partial facial nerve resection.

## Data Availability

The datasets used and/or analysed during the current study are available from the corresponding author on reasonable request.

## Abbreviations

MUP: Motor unit potential
EMG: Electromyographic
